# Dissecting spatiotemporal patterns of functional diversity through the lens of Darwin's naturalization conundrum

**DOI:** 10.1002/ece3.2933

**Published:** 2017-04-21

**Authors:** Sara E. Campbell, Nicholas E. Mandrak

**Affiliations:** ^1^ Department of Ecology and Evolutionary BiologyUniversity of TorontoTorontoONCanada; ^2^ Department of Biological SciencesUniversity of Toronto‐ScarboroughTorontoONCanada

**Keywords:** community assembly, environmental filtering, invasion, niche partitioning, spatial scale, temporal resolution

## Abstract

Darwin's naturalization conundrum describes the paradigm that community assembly is regulated by two opposing processes, environmental filtering and competitive interactions, which predict both similarity and distinctiveness of species to be important for establishment. Our goal is to use long‐term, large‐scale, and high‐resolution temporal data to examine diversity patterns over time and assess whether environmental filtering or competition plays a larger role in regulating community assembly processes. We evaluated Darwin's naturalization conundrum and how functional diversity has changed in the Laurentian Great Lakes fish community from 1870 to 2010, which has experienced frequent introductions of non‐native species and extirpations of native species. We analyzed how functional diversity has changed over time by decade from 1870 to 2010 at three spatial scales (regional, lake, and habitat) to account for potential noninteractions between species at the regional and lake level. We also determined which process, environmental filtering or competitive interactions, is more important in regulating community assembly and maintenance by comparing observed patterns to what we would expect in the absence of an ecological mechanism. With the exception of one community, all analyses show that functional diversity and species richness has increased over time and that environmental filtering regulates community assembly at the regional level. When examining functional diversity at the lake and habitat level, the regulating processes become more context dependent. This study is the first to examine diversity patterns and Darwin's conundrum by integrating long‐term, large‐scale, and high‐resolution temporal data at multiple spatial scales. Our results confirm that Darwin's conundrum is highly context dependent.

## 1 Introduction

Human‐assisted movement has broken down natural barriers to the dispersal of species, drastically increasing both the rate and spatial scale at which biotic exchange occurs (Hulme, 2009; Olden, Poff, Douglas, Douglas, & Fausch, 2004). The introduction of species to areas outside of their native range can have various ecological impacts, especially when acting in synergy with local extirpations to modify the composition, richness, and functioning of communities, effectively altering patterns of biodiversity (Olden & Poff, 2004). Understanding how the assembly of non‐native species contributes to changes in biodiversity, and whether local or regional processes regulate this change, presently comprises one of the central goals to community ecology and conservation (Mcgill, Enquist, Weiher, & Westoby, 2006; Olden et al., 2004).

Species richness does not always adequately reflect patterns of overall diversity, and the processes that regulate species colonization and extirpations are likely better reflected by functional traits, the characteristics that influence the morphology, physiology, phenology, behavior, and life history of species (Díaz et al., 2013; Petchey & Gaston, 2006). Functional diversity refers to the ecological roles that species have in their community and how their traits influence composition and ecosystem functioning (Tilman, 2001). In contrast to strictly assessing taxonomic diversity, utilizing a trait‐based approach provides the ability to address the response of species and communities to anthropogenic stressors and identify the underlying mechanisms of community assembly (Frimpong & Angermeier, 2010).

Darwin was among the first to understand the value of invasive species as a natural experiment for studying community assembly processes. Darwin's naturalization conundrum postulates that community assembly is regulated by two contrasting processes, environmental filtering and competition, which predict both similarity and distinctiveness of species to be important for invasion success (Darwin, 1859). Environmental filtering generally selects for species with similar traits, which ultimately leads to trait convergence, or underdispersion, within communities, while competition often limits the similarity between species due to niche partitioning, effectively leading to trait divergence, or overdispersion within communities (Laughlin, Joshi, van Bodegom, Bastow, & Fulé, 2012). Both environmental filtering and competition can act simultaneously depending on the spatial scale of the observation. Species generally must pass through an environmental filter to persist in a given region, but may experience stronger competitive interactions at finer spatial scales. As non‐native species become introduced, functional diversity will increase, decrease, or remain the same dependent on the uniqueness of the suite of traits of the species introduced. As more unique traits are introduced into a community, one would expect to see an increase in the dispersion of traits and, thus, functional diversity, whereas when species similar to the resident community establish, one would expect to see either no change or a decrease in the dispersion of traits and, thus, functional diversity.

Although changes in biodiversity are pervasive globally, a recent global analysis suggested that, rather than changes in local richness over time, there is a high rate of temporal species turnover, known as β‐diversity, which is directly related to processes of local extirpations and establishment of new species (Dornelas et al., 2014). If β‐diversity decreases over time, this may mean that the community is occupied by similar, closely related, and highly competitive species. When β‐diversity remains the same over time, species may continually colonize and go extinct such that replacement occurs and diversity levels remain at the current level. If β‐diversity increases consistently over time, the community may not yet be at saturation as a result of geographic barriers, dispersal ability of species, insufficient time for colonization, or extreme environmental conditions (Gómez de Silva & Medellín, 2002).

Understanding the processes regulating diversity and the community assembly of species over time requires long‐term data that are generally not available, and those studies that do have temporal replicates are usually on a scale of a few years or decades, or have only completed a comparison of present‐day diversity to that of an historical community (Lindenmayer et al., 2012; Magurran et al., 2010; Willis et al., 2007). Investigations that compare few years or decades of data, often due to limitations of historical data availability or the time interval of the study, lack the temporal resolution to observe fine‐scale patterns of biodiversity and temporal turnover. Furthermore, in the case of evaluating Darwin's naturalization conundrum, patterns may be unnecessarily confounded in snapshot studies without a complete view of how non‐native species change the structure of their novel communities (see Li et al., 2015). When comparing present‐day communities to that of an historical community, it is more difficult to identify the dynamics responsible for the present‐day community, whether competition is more important than environmental filtering, and if the non‐native species that became established are displacing native species within the community given that only two time periods are compared. Additionally, without increased temporal resolution, an ecological pattern may be masked by overall patterns. For example, functional diversity may decrease in two communities at the same point in time, but could be the result of the loss of a unique species in one community and the gain of a similar species in the other community. At a fine scale, the same pattern is occurring for different reasons; however, this may not be apparent when only comparing historical and present‐day communities. When analyzing Darwin's naturalization conundrum, it is necessary to include all stages of invasion and, by increasing temporal replicates, it is more likely that all stages will be included (Li et al., 2015). A fine‐scale temporal resolution is essential to understand how diversity is changing through time, to resolve Darwin's conundrum, and to understand whether the addition of non‐native species has changed the structure of a community over time or if they are displacing native species, allowing the community structure to remain the same.

Although changes in taxonomic diversity have been well‐documented over time, less is known about how other diversity metrics may change temporally and changes in taxonomic diversity may not necessarily reflect the underlying patterns and processes (Dreiss et al., 2015; Villéger, Grenoillet, & Brosse, 2014). Here, based on literature on temporal intervals (see Bengtsson, Baillie, & Lawton, 1997; Diamond & May, 1977; Russell, Diamond, Pimm, & Reed, 1995), we determined the most appropriate temporal scale necessary to understand community dynamics and examined changes in functional diversity on a decadal scale, 1870–2010, in Laurentian Great Lakes fish communities that have experienced frequent and well‐documented introductions of non‐native species and extirpation of native species. To the best of our knowledge, no study has examined long‐term patterns of diversity with data spanning over a century at a temporal scale that has a high power of resolution.

In addition, most previous studies have examined patterns at a regional or community level, such that some species will not interact or compete with each other (Li et al., 2015). We assessed differences in functional diversity at the various spatial scales (i.e., regional, community, habitat); thus, accounting for whether species share biotic interactions and, in doing so, also disentangle the relative contribution of different habitats to overall diversity patterns. In addition, when analyzing Darwin's naturalization conundrum, we also identify whether the dominant regulating process is dependent upon the spatial scale at which the study is completed.

The most limiting factor in studying temporal patterns of diversity has been identified as the availability of long‐term, large‐scale, and high‐resolution data (Dornelas et al., 2012). Here, we have high temporal replicates and examine patterns of diversity at multiple spatial scales to understand and identify how diversity is changing through time and what is driving the patterns, which ultimately allows us to evaluate Darwin's naturalization conundrum using the Laurentian Great Lakes as a study system. We expect that our ability to interpret Darwin's conundrum will be more informative at smaller spatial scales where species are interacting (i.e., at the habitat level) and that, through identifying the most appropriate temporal scale, we will gain high temporal resolution, which will provide the first evidence of long‐term diversity dynamics with the ability to also understand how diversity changes in short time‐steps in response to the addition of non‐native species and extirpation of native species. This study is the first to comprehensively integrate long‐term, large‐scale, and high‐resolution temporal data and multiple spatial scales to analyze diversity patterns over time and to evaluate Darwin's conundrum.

## 2 Methods

### 2.1 Datasets

Fish occurrences in the Laurentian Great Lakes basin (Lake Erie, Lake Huron, Lake Michigan, Lake Ontario, Lake Superior) were compiled by decade from 1870 to present (2010) based on a Great Lakes species list (Roth, Mandrak, Hrabik, Sass, & Peters, 2013) and lists of introduced and extirpated species (Mandrak & Cudmore, 2010, 2013). This dataset includes 182 freshwater fish species, with 150 native species, three established and possibly native species, 29 introduced and established non‐native species, with 15 extirpations and four extinctions of native species (Roth et al., 2013). Additionally, as species occupy different habitat types and may not necessarily interact with all other species present in the community in a given decade, the species present in each lake were further partitioned into different habitat types (i.e., Great Lakes Offshore, Great Lakes Nearshore, Great Lakes Wetlands, Inland Lakes, Inland Rivers) using current habitat preferences from the literature (Eakins, 2015; Froese & Pauly, 2015; Holm, Mandrak, & Burridge, 2009; Hubbs & Lagler, 2004; Jude & Pappas, 1992; Mandrak & Crossman, 1992; Seilheimer & Chow‐Fraser, 2007; Trebitz, Brazner, Brady, Axler, & Tanner, 2007; Trebitz & Hoffman, 2015). A species trait database for all Great Lakes freshwater fishes was compiled based on existing databases, and the functional diversity of each species was calculated based on 12 functional traits commonly used in studies on fish functional diversity (Coker, Portt, & Minns, 2001; Eakins, 2015; Frimpong & Angermeier, 2009; Froese & Pauly, 2015). These traits include maximum length, length at first reproduction, age at first reproduction, longevity, fecundity, egg diameter, length at hatch, Balon guild, spawning depth, feeding depth, and diet breadth (Table 1; Olden, Poff, & Bestgen, 2006; Frimpong & Angermeier, 2010; Villéger et al., 2014). Trait values are in the form of ordinal, ranked ordinal, or continuous data (Table 1). For analyses, continuous traits with a range were assigned median values. A diet breadth index, scored 1–9, was developed based on the number of prey items for which each species had a medium or high preference throughout their life (Table 1). A spawning substrate breadth index, scored 1–10, was developed based on the number of substrates for which each species had a high or medium preference during spawning (Table 1). For species with trait values not present in any of these databases, values were taken from other sources for populations geographically closest to the Great Lakes where possible (see Appendix S1 in Supporting Information, Table S1).

**Table 1:** Species traits used in FDis analysis. For spawning and feeding depth, values go from high (1) to no preference (4). For spawning substrate breadth, values range from 1, which corresponds to a specialist, to 10, which corresponds to a generalist. Diet breadth was analyzed the same way, where a 1 corresponds to a specialist and a 9 corresponds to a generalist

Trait	Data type	Range (if ordinal)
Maximum length	Continuous	
Length at first reproduction	Continuous	
Age at maturation	Continuous	
Longevity	Continuous	
Fecundity	Continuous	
Egg diameter	Continuous	
Length at hatch	Continuous	
Balon guild	Ordinal	1–14
Spawning depth	Ranked ordinal	1–4
Spawning substrate breadth	Ranked ordinal	1–10
Feeding depth	Ranked ordinal	1–4
Diet breadth	Ranked ordinal	1–9

### 2.2 Data analyses

Functional diversity was calculated using functional dispersion (FDis), which calculates the mean distance of each species to the centroid of an ordination plot of the first three axes of all species within the community and allows for both missing data and mixed variables (Laliberté & Legendre, 2010). Previous studies have shown that β‐diversity is underestimated when completing analyses at intervals of a decade or more (Diamond & May, 1977; Russell et al., 1995); thus, to analyze fine‐scale patterns and maintain high temporal resolution, we used a 10‐year temporal interval for all analyses. FDis was calculated for the regional source pool by decade and was calculated for each lake by decade. To determine whether functional space had increased, decreased, or remained the same over time regionally (basin), by community (lake), and locally (habitat), differences in FDis were calculated between: i) each time period and the previous time period; and, ii) each time period and 1870. For each decade, the mean FDis for species present in each habitat type was calculated, and a species could occupy more than one habitat type depending on its habitat preferences as an adult.

To evaluate whether the observed patterns of functional diversity are more or less extreme than expected in the absence of an ecological mechanism, a null model was constructed for the basin and each lake. We completed a randomization simulation by decade on the species by trait matrix by randomly selecting species without replacement from the regional species pool, such that species richness was held constant between the observed and simulated communities. Additionally, at the lake level, the time of arrival for each invasive species was constrained so each invasive species could be selected only once the opportunity for establishment and dispersal was possible. For each decade at the basin and lake level, we calculated mean FDis for each randomization and completed this for a total of 1,000 times; we then calculated the overall mean, 95% confidence interval (CI), and standard error. This enabled us to determine whether an ecological mechanism, competition or environmental filtering, regulates diversity patterns in the Great Lakes, giving us the ability to analyze Darwin's naturalization conundrum. Observed values of FDis above the upper threshold of the 95% CI indicate that species are more overdispersed than expected under the null model, which suggests that competitive interactions are more important in regulating diversity patterns, whereas observed values of FDis below the lower threshold of the 95% CI indicate that species are more underdispersed than expected under the null model, suggesting that an environmental filter regulates diversity patterns and selects for more similar species. All analyses were completed using the FD package in R (Laliberté & Legendre, 2010).

## 3 Results

### 3.1 Species richness and functional diversity

**Figure 1:**
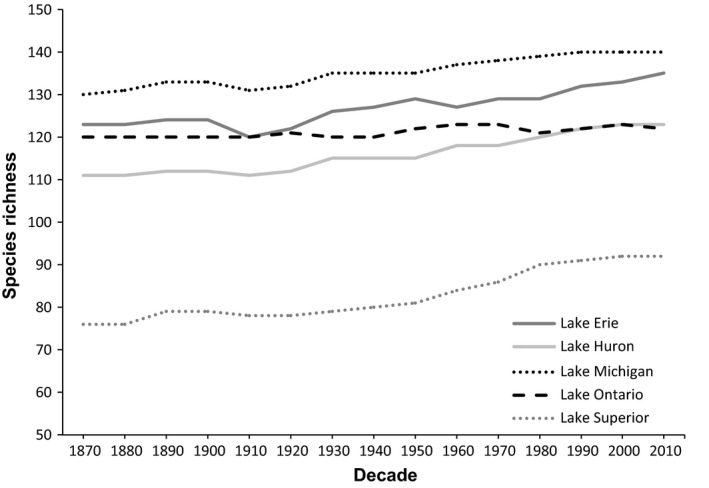
Species richness by decade through time for Lake Erie, Lake Huron, Lake Michigan, Lake Ontario, and Lake Superior

**Figure 2:**
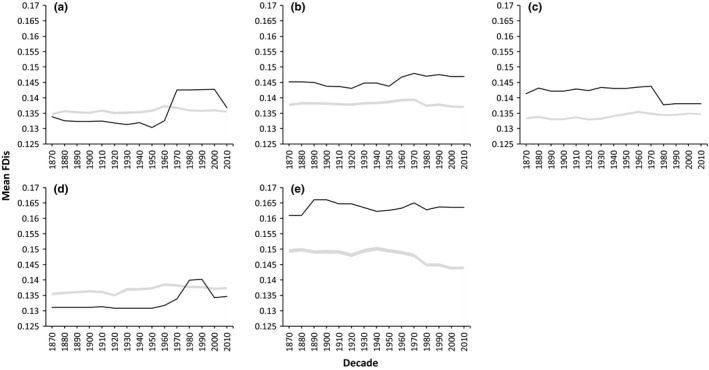
Mean functional diversity (FDis) by decade through time for (a) Lake Erie, (b) Lake Huron, (c) Lake Michigan, (d) Lake Ontario, and (e) Lake Superior where the solid black line is the observed FDis through time, and the shaded gray area corresponds to the mean and 95% confidence interval (CI) of the null model. Because the 95% CI is tightly concentrated around the mean, the shaded area appears as a line in some cases

Our results show that levels of functional diversity generally do not coincide with species richness (Figures 1 and 2). Lake Superior has the lowest species richness of all the lakes, with 95 species present, 75 of which are native; however, it has a markedly higher mean functional diversity in comparison with the other lakes. In contrast, Lake Michigan has the highest species richness, with 149 total species, 126 being native, and fluctuates among the bottom three lakes over time for mean functional diversity. When accounting for introductions, Lake Superior had proportionally the highest percentage of non‐native species that successfully established and the lowest percentage of native species that became extirpated. Lake Ontario, which generally had the lowest mean functional diversity of the five lakes, has 131 total species, 110 of which are native, and proportionally has the lowest percentage of non‐native species that successfully established. Overall, where one lake ranks for species richness is not indicative of its level of mean functional diversity in relation to the other four lakes (Figures 1 and 2).

### 3.2 Patterns of functional diversity

**Figure 3:**
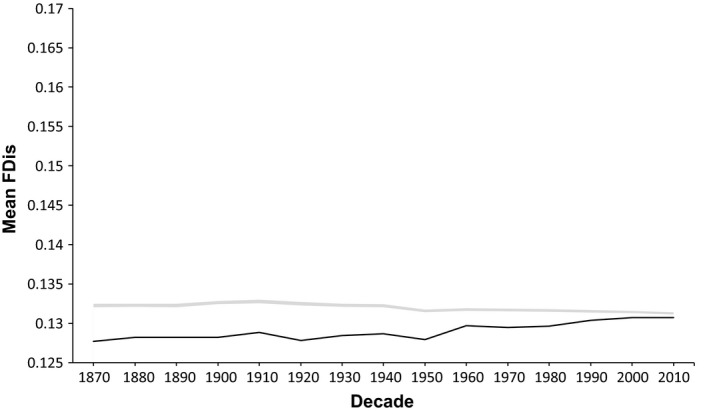
Mean functional diversity (FDis) by decade through time for the regional source pool, where the solid black line corresponds to actual FDis and the shaded gray area corresponds to the mean and 95% confidence interval (CI) of the null model. Because the 95% CI is concentrated around the mean, the shaded area appears as a line

Through the completion of randomization simulations, we found that the actual mean functional diversity in any given decade at the regional spatial scale was lower than expected under the null model; therefore, the Great Lakes fish communities exhibited underdispersion (Figure 2). At the lake level, patterns are context dependent given that the observed mean functional diversity was lower than expected under the null model in Lake Erie and Lake Ontario, demonstrating communities are underdispersed, whereas in Lake Huron, Lake Michigan, and Lake Superior, the observed functional diversity was higher than expected under the null model, demonstrating that these communities are overdispersed (Figure 3). There is a general increasing trend in the observed mean over time, with the exception of Lake Michigan. Between decades, functional diversity fluctuates as non‐native species become established and native species become extirpated. When comparing the present community (2010) to that of the historical community (1870), we find that, with the exception of Lake Michigan, each lake has increased in mean functional diversity. When comparing communities between each decade and the historical period, trends become more complex with some decreases in response to the loss of native species. Of all the lakes, Lake Michigan is the only lake to consistently show a negative trend in functional diversity throughout all analyses. When considering the regional pool, we find that functional diversity fluctuates between decades as non‐native species are introduced and native species are extirpated, and when comparing the present community to that of the historical community, there has been an increase in functional diversity regionally, suggesting that the suite of traits introduced by the non‐native species are unique to the region.

### 3.3 Functional diversity by habitat

**Figure 4:**
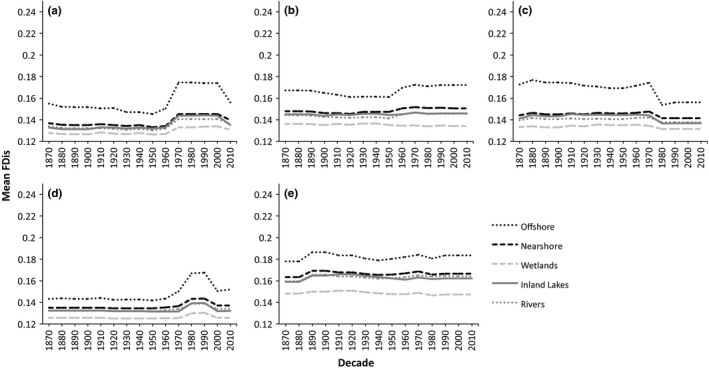
Functional diversity by decade through time for each habitat in (a) Lake Erie, (b) Lake Huron, (c) Lake Michigan, (d) Lake Ontario, and (e) Lake Superior

When comparing functional diversity among habitats in each lake (Figure 4), the Great Lakes offshore fish community consistently has the highest mean functional diversity through time, while Great Lakes wetlands community has the lowest. Great Lakes nearshore communities always have the second highest mean functional diversity across all lakes. Inland lake and river communities often have very similar means for functional diversity. With the exception of Lake Michigan, functional diversity in each lake for all habitats has a slight increase, with the most dramatic changes occurring within the past six decades. Lake Superior, which has the highest functional diversity of all lakes, also generally had a higher mean functional diversity for all habitat types in comparison with the other four lakes, whereas mean functional diversity for habitat types in Lake Ontario is generally lower in comparison with the other four lakes.

## 4 Discussion

Although we have found increases in species richness and functional diversity in the Laurentian Great Lakes, the majority of studies show that non‐native species pose a large threat to native community biodiversity (Powell, Chase, & Knight, 2013; Sala et al., 2000; Vilà et al., 2011). Our results have shown that the addition of non‐native species in the Great Lakes has increased functional diversity, at both regional and lake levels over the past 140 years and appears to be primarily regulated by environmental filtering at a regional spatial scale; however, the ability to evaluate Darwin's conundrum is dependent on the spatial scale at which a study is completed. We found that even at the lake level, determining the dominant regulating process was still context dependent, suggesting that studies completed at large spatial scales are unlikely to definitively resolve Darwin's conundrum; there may be no general consensus among studies even at smaller spatial scales and resolving Darwin's conundrum is likely highly case specific.

Although species richness decreases from south to north in the Great Lakes basin (Staton & Mandrak, 2005), patterns of functional diversity do not follow the same latitudinal trend, which has already been shown, latitudinal or otherwise, for taxonomic diversity in other systems (Devictor et al., 2010; Lamanna et al., 2014; Monnet et al., 2014; Villéger et al., 2014). Lake Michigan has the highest species richness but the lowest mean functional diversity over time, while Lake Superior has the lowest species richness but highest mean functional diversity over time. As many species in Lake Michigan are at the northern limit of their geographic range (Page & Burr, 2011), the species present need to surpass a climatic barrier to disperse to environmentally suitable portions of other lakes. Lake Superior may have higher functional diversity in comparison with the other five lakes for various reasons; species in Lake Superior may experience a strong level of niche partitioning or, perhaps because Lake Superior is the largest and deepest of the five lakes, it may have unique species due to the types of habitats present, such as the six coregonine species present (Roth et al., 2013). These results highlight the importance of examining multiple metrics of biodiversity when studying changes in patterns over time.

When solely examining patterns of functional diversity, we found that environmental filtering may play a large role in shaping species assembly within the Great Lakes at the regional level, while both environmental filtering and competitive interactions are important at the lake and habitat level where species are more likely to interact. Low levels of functional diversity in relation to what we expect under the null model suggests that species in the Great Lakes are underdispersed in their traits, or are clustering, which indicates that an environmental filter may limit species dissimilarity, both regionally and in Lake Erie and Lake Ontario, due to environmental conditions. As species are more similar in the functional space they occupy, we expect that environmental conditions may be severe enough that the filter persists as a barrier to establishment and that the temporal increase in functional diversity is a function of human‐mediated dispersal of non‐native species (Mandrak & Cudmore, 2010). This could explain the trends present in Lake Michigan; Lake Michigan has the highest species richness but lowest functional diversity and was the only lake to see a consistent decrease in mean functional diversity over time despite proportionally losing and gaining the same percentage of species as Lake Erie and Lake Huron. This suggests that the functional traits of the species lost, such as the Kiyi (*Coregonus kiyi*) or Paddlefish (*Polyodon spathula*), were unique within the community, or the species gained, such as the Goldfish (*Carassius auratus*) and Redear Sunfish (*Lepomis microlophus*), were similar to species already in the community due to a shared affinity for local environmental conditions, causing more clustering. As β‐functional diversity exhibited both increasing and decreasing trends, and the observed functional diversity was higher than expected under the null model, the community is likely occupied by similar, closely related, and highly competitive species, which aligns well with the hypothesis that environmental filtering is regulating diversity patterns regionally, but at the lake level, competitive interactions are more important.

Understanding patterns of diversity, community assembly, and coexistence of species, although typically studied at a regional or community levels as discussed above, are best viewed at smaller spatial scales, at which species frequently interact and potentially compete with each other. At a large spatial scale, species may not necessarily interact and, thus, diversity and coexistence patterns may be incorrectly interpreted, whereas at smaller spatial scales, the role of biotic interactions as a driving mechanism of prevalent trends can be examined (Jiang, Tan, & Pu, 2010). Through partitioning species into different habitats, we accounted for noninteraction between species, directly tested how species contribute to overall diversity, and examined how species in different habitats contribute differently to the loss and gain of functional diversity over time.

When examining Darwin's naturalization conundrum, we found that there is context dependence even at smaller spatial scales at which species are potentially interacting. When assessed in conjunction with each other, numerous studies show that interpreting Darwin's conundrum is context dependent at larger spatial scales (Cadotte, Hamilton, & Murray, 2009; Carboni et al., 2013; Li et al., 2015; Thuiller et al., 2010), where all species in a community are unlikely to interact. However, we have shown here that the processes regulating diversity vary even at the habitat level where species present are likely to have interactions. The mean functional diversity for species found in wetlands is the lowest, whereas it was highest for species found in offshore habitats. Regionally, wetlands generally have a higher number of species because they are more diverse habitats (Jude & Pappas, 1992); furthermore, species may not necessarily be resident species of wetlands, but may be migratory species that utilize wetland habitat only for spawning, nursery areas, refuge from predation, or food (Jude & Pappas, 1992; Trebitz & Hoffman, 2015). Thus, functional diversity may be lowest due to an environmental filter present in wetlands, such as vegetation and higher temperatures, but not in offshore habitat (Jude & Pappas, 1992; Trebitz & Hoffman, 2015). Species that utilize wetlands may have similar suites of traits to account for environmental conditions, which will cause an underdispersion of traits and, thus, a lower mean functional diversity. Regionally, of the 182 species present, only 54 utilize offshore habitat; yet, this habitat has the highest mean functional diversity, suggesting that species utilizing this habitat have more unique traits and, thus, are overdispersed indicating that competition plays a larger role in regulating species coexistence in that habitat. Here, we showed that, although environmental filtering is likely driving patterns of functional diversity regionally, when examining smaller spatial scales where species are actually interacting, the patterns vary drastically and are more context dependent, which was also evident at the lake level.

By examining patterns at the decadal level, we were able to observe fine‐scale patterns and temporal turnover; overall, we found that functional diversity is increasing but, between decades, there are sharp increases and declines in diversity levels that correspond to the addition and loss of species. Without this resolution, we would only see the end result of an increasing trend and miss the pattern of loss of unique native species and subsequent replacement by non‐native species between decades.

Our study demonstrates that patterns of species richness do not coincide with functional diversity and that, in the Laurentian Great Lakes, regional‐level diversity patterns may be a function of environmental filtering rather than competition. We also show that spatial scale is important in understanding Darwin's naturalization conundrum and that, even at smaller spatial scales where we expect species interactions to occur, patterns of coexistence are still context dependent. Given that both increases and decreases in functional diversity occurred from decade to decade at all spatial scales, but a general increasing trend was present over time when comparing the historical community to the present‐day community studies, analyzing diversity patterns within a few decades may not be a long enough time series to fully understand diversity changes over time and studies that compare historical data to present day may miss substantial changes between decades in response to the addition of non‐native species and/or extirpation of native species. Our study highlights the need and importance for both long‐time series data and further distinguishing the relative importance of competition and environmental filtering for community assembly, coexistence patterns, and diversity trends over time in the future studies.

## Author contributions

S.E.C. co‐conceived the question and methodological approach, conducted the analysis, and wrote the manuscript. N.E.M. co‐conceived the question and methodological approach, obtained funding, provided data, and edited the manuscript.
