# Supplementary material for: Dissecting spatiotemporal patterns of functional diversity through the lens of Darwin's naturalization conundrum
Source: Ecol Evol. 2017 Apr 21;7(11):3861–9. doi: 10.1002/ece3.2933 (PMC5468161; doi:10.1002/ece3.2933)
Supplement: Supplementary file 1 [file ECE3-7-3861-s001.docx]

**Supporting Information- Appendix 1**

**Table S1.** Reference list for trait values found in the literature for populations geographically closest to the Great Lakes where possible.

| **Species** | **Trait** | **Reference** |
| --- | --- | --- |
| *Acantharcus pomotis* | Egg Diameter | Cooke, S.J. & Philip, D.P. (2009). *Centrarchid Fishes: Diversity, Biology, and Conservation.* Wiley-Blackwell, West Sussex, U.K. |
| *Alosa chrysochloris* | Egg Diameter | Lee, D.S., Gilbert, C.R., Hocutt, C.H., Jenkins, R.E., McAllister, D.E. & Stauffer, J.R. (1980). *Atlas of North American Freshwater Fishes.* North Carolina State Museum of Natural History, Raleigh, N.C., USA. |
|  | Balon Guild | Simon, T.P. (1998). Assessing the Sustainability and Biological Integrity of Water Resources Using Fish Communities*.* CRC Press. 108. |
| *Ambloplites ariommus* | Egg Diameter | Cooke, S.J. & Philip, D.P. (2009). *Centrarchid Fishes: Diversity, Biology, and Conservation.* Wiley-Blackwell, West Sussex, U.K. |
| *Ameiurus catus* | Egg Diameter | Simon, T.P. & Wallus, R. (2004). *Reproductive Biology and Early Life History of Fishes in the Ohio River Drainage, Volume 3: Ictaluridae- Catfish and Madtoms.* CRC Press, New York, New York, USA. |
| *Ammocrypta clara* | Egg Diameter | Carlander, K.D. (1997). *Handbook of Freshwater Fishery Biology - Volume 3: Life History Data on Ichtyopercid Fishes of the United States and Canada.* Iowa State University Press, Ames, USA. |
| *Ammocrypta pellucida* | Egg Diameter | Finch, M., Faber, J.E., Koops, M.A., Dok, S.A. & Power, M. (2013). Biological traits of eastern sand darter (*Ammocrypta pellucida*) in the lower Thames River, Canada, with comparisons to a more southern population. *Ecology of Freshwater Fish,* **76**, 234-245. |
| *Anguilla rostrata* | Age at Maturation | Carlander, K.D. (1969). *Handbook of Freshwater Fishery Biology - Volume 1: Life History Data on Freshwater Fishes of the United States and Canada, Exclusive of the Perciformes*. Iowa State University Press, Ames, USA.  Etnier, D.A. & Starnes, W.C. (1993). *The Fishes of Tennessee*. University of Tennessee Press, Knoxville, Tennessee, USA. |
|  | Fecundity | Carlander, K.D. (1969). *Handbook of Freshwater Fishery Biology - Volume 1: Life History Data on Freshwater Fishes of the United States and Canada, Exclusive of the Perciformes.* Iowa State University Press, Ames, USA.  Etnier, D.A., & Starnes, W.C. (1993). *The Fishes of Tennessee*. University of Tennessee Press, Knoxville, Tennessee, USA. |
| *Campostoma oligolepis* | Egg Diameter | Coburn, M.M. (1986). Egg diameter variation in eastern North American minnows (*Pisces: Cyprinidae*): Correlation with vertebral number, habitat and spawning behavior. *Ohio Journal of Science,* **86**, 110-120. |
| *Carpiodes carpio* | Length at Maturation | Trautman, M.B. (1981). *The Fishes of Ohio*. Second Edition. Ohio State University Press, Columbus, USA. |
|  | Longevity | Carlander, K.D. (1969). *Handbook of Freshwater Fishery Biology - Volume 1: Life History Data on Freshwater Fishes of the United States and Canada, Exclusive of the Perciformes*. Iowa State University Press, Ames, USA.  Etnier, D.A. & Starnes, W.C. (1993). *The Fishes of Tennessee*. University of Tennessee Press, Knoxville, Tennessee, USA. |
|  | Egg Diameter | Mitton, J.B. & Lewis Jr., W.M. (1989). Relationships between genetic variability and life-history features of bony fishes. *Evolution*, **43**, 1712-1723. |
|  | Balon Guild | Simon, T.P. (1998). Assessing the Sustainability and Biological Integrity of Water Resources Using Fish Communities*.* CRC Press. 108. |
|  | Spawning Depth | Kay, L.K., Wallus, R. & Yeager, B.L. (1994). *Reproductive Biology and Early Life History of Fishes in the Ohio River Drainage. Vol. 2. Catostomidae.* Tennessee Valley Authority, Chattanooga, Tennessee, USA. |
|  | Spawning Substrate | Kay, L.K., Wallus, R. & Yeager, B.L. (1994). *Reproductive Biology and Early Life History of Fishes in the Ohio River Drainage. Vol. 2. Catostomidae.* Tennessee Valley Authority, Chattanooga, Tennessee, USA. |
|  | Diet Breadth | Robinson, W.R. & Buchanan, T.M. (1992). *Fishes of Arkansas.* The University of Arkansas Press, Fayetteville, Arkansas. USA. |
| *Cyprinella lutrensis* | Egg Diameter | Saksena, V.P. (1962). The post-hatching stages of the Red Shiner, *Notropis lutrensis*. *Copeia*, **3**,539-544. |
| *Enneacanthus chaetodon* | Egg Diameter | Cooke, S.J. & Philip, D.P. (2009). *Centrarchid Fishes: Diversity, Biology, and Conservation.* Wiley-Blackwell, West Sussex, U.K. |
| *Enneacanthus gloriosus* | Egg Diameter | Cooke, S.J. & Philip, D.P. (2009). *Centrarchid Fishes: Diversity, Biology, and Conservation.* Wiley-Blackwell, West Sussex, U.K. |
| *Enneacanthus obesus* | Egg Diameter | Cooke, S.J. & Philip, D.P. (2009). *Centrarchid Fishes: Diversity, Biology, and Conservation.* Wiley-Blackwell, West Sussex, U.K. |
| *Erimyzon oblongus* | Egg Diameter | Fuiman, L.A. (1979). Descriptions and comparisons of *Castomid* fish larvae: Northern Atlantic drainage species. *Transactions of the American Fisheries Society*, **108**, 560-603. |
| *Esox americanus vermiculatus* | Age at Maturation | Carlander, K.D. (1969). *Handbook of Freshwater Fishery Biology - Volume 1: Life History Data on Freshwater Fishes of the United States and Canada, Exclusive of the Perciformes*. Iowa State University Press, Ames, USA. |
|  | Fecundity | Carlander, K.D. (1969). *Handbook of Freshwater Fishery Biology - Volume 1: Life History Data on Freshwater Fishes of the United States and Canada, Exclusive of the Perciformes*. Iowa State University Press, Ames, USA. |
| *Esox niger* | Egg Diameter | Scott, W.B. & Crossman, E.J. (1973). *Freshwater Fishes of Canada.* Bulletin 184. Fisheries Research Board of Canada, Ottawa, Canada. |
|  | Length at Hatch | Scott, W.B. & Crossman, E.J. (1973). *Freshwater Fishes of Canada.* Bulletin 184. Fisheries Research Board of Canada, Ottawa, Canada. |
| *Etheostoma chlorosomum* | Egg Diameter | Carlander, K.D. (1997). *Handbook of Freshwater Fishery Biology - Volume 3: Life History Data on Ichtyopercid Fishes of the United States and Canada.* Iowa State University Press, Ames, USA. |
| *Exoglossum laurae* | Egg Diameter | Carlander, K.D. (1969). *Handbook of Freshwater Fishery Biology - Volume 1: Life History Data on Freshwater Fishes of the United States and Canada, Exclusive of the Perciformes*. Iowa State University Press, Ames, USA.  Smith, C.L. (1985). *The Inland Fishes of New York State*. New York State Department of Environmental Conservation, Albany, USA. |
| *Fundulus catenatus* | Egg Diameter | Fisher, J.W. (1981). Ecology of *Fundulus catenatus* in three interconnected stream orders. *The American Midland Naturalist Journal*, **106**, 372-378. |
| *Fundulus dispar* | Balon Guild | Simon, T.P. (1998). Assessing the Sustainability and Biological Integrity of Water Resources Using Fish Communities*.* CRC Press. 108. |
| *Hiodon alosoides* | Longevity | Carlander, K.D. (1969). *Handbook of Freshwater Fishery Biology - Volume 1: Life History Data on Freshwater Fishes of the United States and Canada, Exclusive of the Perciformes*. Iowa State University Press, Ames, USA. |
| *Hybognathus regius* | Egg Diameter | Raney, E.C. (1939). The breeding habits for the silvery minnow, *Hybognathus regius* Girard. *The American Midland Naturalist Journal*, **21**, 674-680. |
| *Hypentelium nigricans* | Egg Diameter | Fuiman, L.A. (1979). Descriptions and comparisons of *Castomid* fish larvae: Northern Atlantic drainage species. *Transactions of the American Fisheries Society*, **108**, 560-603. |
| *Ictiobus bubalus* | Egg Diameter | Ross, S.T. (2001). *The Inland Fishes of Mississippi.* Mississippi Department of Wildlife, Fisheries and Parks. Singapore. |
|  | Length at Hatch | Ross, S.T. (2001). *The Inland Fishes of Mississippi.* Mississippi Department of Wildlife, Fisheries and Parks. Singapore. |
| *Ictiobus niger* | Egg Diameter | Kay, L.K., Wallus, R. & Yeager B.L. (1994). *Reproductive Biology and Early Life History of Fishes in the Ohio River Drainage. Vol. 2. Catostomidae.* Tennessee Valley Authority, Chattanooga, Tennessee, USA. |
| *Lepomis humilis* | Egg Diameter | Mitton, J.B. & Lewis Jr., W.M. (1989). Relationships between genetic variability and life-history features of bony fishes. *Evolution*, **43**, 1712-1723. |
| *Lepomis punctatus* | Length at Maturation | Cooke, S.J. & Philip, D.P. (2009). *Centrarchid Fishes: Diversity, Biology, and Conservation.* Wiley-Blackwell, West Sussex, U.K. |
| *Lythrurus ardens* | Egg Diameter | Coburn, M.M. (1986). Egg diameter variation in eastern North American minnows (*Pisces: Cyprinidae*): correlation with vertebral number, habitat and spawning behavior. *Ohio Journal of Science*, **86**, 110-120. |
| *Macrhybopsis storeriana* | Egg Diameter | Coburn, M.M. (1986). Egg diameter variation in eastern North American minnows (*Pisces: Cyprinidae*): correlation with vertebral number, habitat and spawning behavior. *Ohio Journal of Science*, **86**, 110-120. |
| *Minytrema melanops* | Egg Diameter | Kay, L.K., Wallus, R. & Yeager B.L. (1994). *Reproductive Biology and Early Life History of Fishes in the Ohio River Drainage. Vol. 2. Catostomidae.* Tennessee Valley Authority, Chattanooga, Tennessee, USA. |
| *Misgurnus anguillicaudatus* | Length at Hatch | Gao, L., Duan, M., Cheng, F. & Xie, S. (2014). Ontogenetic development in the morphology and behavior of loach (*Misgurnus anguillicaudatus*) during early life stages. *Chinese Journal of Oceanology and Limnology*, **32**, 973-981. |
| *Notropis amblops* | Egg Diameter | Coburn, M.M. (1986). Egg diameter variation in eastern North American minnows (*Pisces: Cyprinidae*): correlation with vertebral number, habitat and spawning behavior. *Ohio Journal of Science*, **86**, 110-120. |
| *Notropis blennius* | Egg Diameter | Coburn, M.M. (1986). Egg diameter variation in eastern North American minnows (*Pisces: Cyprinidae*): correlation with vertebral number, habitat and spawning behavior. *Ohio Journal of Science*, **86**, 110-120. |
| *Notropis boops* | Egg Diameter | Coburn, M.M. (1986). Egg diameter variation in eastern North American minnows (*Pisces: Cyprinidae*): correlation with vertebral number, habitat and spawning behavior. *Ohio Journal of Science*, **86**, 110-120. |
| *Notropis buccatus* | Egg Diameter | Hoyt, R.D. (1971). The reproductive biology of the Silverjaw Minnow, *Ericymba buccata* Cope, in Kentucky. *Transactions of the American Fisheries Society*, **100**, 510-519. |
| *Notropis buchanani* | Egg Diameter | Coburn, M.M. (1986). Egg diameter variation in eastern North American minnows (*Pisces: Cyprinidae*): correlation with vertebral number, habitat and spawning behavior. *Ohio Journal of Science*, **86**, 110-120. |
| *Notropis chalybaeus* | Egg Diameter | Carlander, K.D. (1969). *Handbook of Freshwater Fishery Biology - Volume 1: Life History Data on Freshwater Fishes of the United States and Canada, Exclusive of the Perciformes*. Iowa State University Press, Ames, USA.  Smith, C.L. (1985). *The Inland Fishes of New York State*. New York State Department of Environmental Conservation, Albany, USA. |
| *Notropis heterodon* | Egg Diameter | Keast, A. & Eadie, J. (1984). Growth in the first summer of life: a comparison of nine co-occurring fish species. *Canadian Journal of Zoology*, **62**, 1242-1250. |
| *Notropis nubilis* | Egg Diameter | Coburn, M.M. (1986). Egg diameter variation in eastern North American minnows (*Pisces: Cyprinidae*): correlation with vertebral number, habitat and spawning behavior. *Ohio Journal of Science*, **86**, 110-120. |
| *Notropis procne* | Egg Diameter | Olmstead, L.L. (1977). *Proceedings of the First Symposium on Freshwater Larval Fish*. Charlotte, N.C., USA. |
| *Notropis texanus* | Egg Diameter | Carlander, K.D. (1969). *Handbook of Freshwater Fishery Biology - Volume 1: Life History Data on Freshwater Fishes of the United States and Canada, Exclusive of the Perciformes*. Iowa State University Press, Ames, USA. |
| *Noturus exilis* | Longevity | Etnier, D.A. & Starnes, W.C. (1993). *The Fishes of Tennessee*. University of Tennessee Press, Knoxville, USA. |
| *Noturus insignis* | Egg Diameter | Simon, T.P. & Wallus, R. (2004). *Reproductive Biology and Early Life History of Fishes in the Ohio River Drainage, Volume 3: Ictaluridae- Catfish and Madtoms.* CRC Press, New York, New York, USA. |
| *Noturus leptacanthus* | Egg Diameter | Carlander, K.D. (1969). *Handbook of Freshwater Fishery Biology - Volume 1: Life History Data on Freshwater Fishes of the United States and Canada, Exclusive of the Perciformes*. Iowa State University Press, Ames, USA.  Etnier, D.A. & Starnes, W.C. (1993). *The Fishes of Tennessee*. University of Tennessee Press, Knoxville, USA. |
| *Noturus nocturnus* | Longevity | Carlander, K.D. (1969). *Handbook of Freshwater Fishery Biology - Volume 1: Life History Data on Freshwater Fishes of the United States and Canada, Exclusive of the Perciformes*. Iowa State University Press, Ames, USA.  Etnier, D.A. & Starnes, W.C. (1993). *The Fishes of Tennessee*. University of Tennessee Press, Knoxville, USA. |
| *Myoxocephalus thompsoni* | Egg Diameter | Jacoby, C. (1953). *Notes on the life history of the Deepwater Sculpin, Myoxocephalus quadricornis L., in Lake Superior*. Ann Arbor, Michigan: Department of Fisheries, School of Natural Resources, University of Michigan. |
| *Percina evides* | Egg Diameter | Carlander, K.D. (1997). *Handbook of Freshwater Fishery Biology - Volume 3: Life History Data on Ichtyopercid Fishes of the United States and Canada.* Iowa State University Press, Ames, USA. |
| *Percina shumardi* | Egg Diameter | Carlander, K.D. (1997). *Handbook of Freshwater Fishery Biology - Volume 3: Life History Data on Ichtyopercid Fishes of the United States and Canada.* Iowa State University Press, Ames, USA. |
| *Phenacobius mirabilis* | Egg Diameter | Coburn, M.M. (1986). Egg diameter variation in eastern North American minnows (*Pisces: Cyprinidae*): correlation with vertebral number, habitat and spawning behavior. *Ohio Journal of Science*, **86**, 110-120. |
| *Phoxinus erythrogaster* | Egg Diameter | Coburn, M.M. (1986). Egg diameter variation in eastern North American minnows (*Pisces: Cyprinidae*): correlation with vertebral number, habitat and spawning behavior. *Ohio Journal of Science*, **86**, 110-120. |
| *Pimephales vigilax* | Egg Diameter | Coburn, M.M. (1986). Egg diameter variation in eastern North American minnows (*Pisces: Cyprinidae*): correlation with vertebral number, habitat and spawning behavior. *Ohio Journal of Science*, **86**, 110-120. |
| *Platichthys flesus* | Maximum Length | Berg, L.S. (1964). *Freshwater Fishes of the USSR and Adjacent Countries*. 4th edition. Israel Program for Scientific Translation, Jerusalem.  Wheeler, A. (1969). *Fishes of the British Isles and Northwest Europe.* Michigan State University Press, East Lansing, USA.  Maitland, P.S. (2001). *Guide to Freshwater Fish of Britain and Europe.* Hamlyn, London, UK. |
|  | Age at Maturation | Berg, L.S. (1964). *Freshwater Fishes of the USSR and Adjacent Countries*. 4th edition. Israel Program for Scientific Translation, Jerusalem.  Wheeler, A. (1969). *Fishes of the British Isles and Northwest Europe.* Michigan State University Press, East Lansing, USA.  Maitland, P.S. (2001). *Guide to Freshwater Fish of Britain and Europe.* Hamlyn, London, UK. |
|  | Longevity | Berg, L.S. (1964). *Freshwater Fishes of the USSR and Adjacent Countries*. 4th edition. Israel Program for Scientific Translation, Jerusalem.  Wheeler, A. (1969). *Fishes of the British Isles and Northwest Europe.* Michigan State University Press, East Lansing, USA.  Maitland, P.S. (2001). *Guide to Freshwater Fish of Britain and Europe.* Hamlyn, London, UK. |
|  | Fecundity | Berg, L.S. (1964). *Freshwater Fishes of the USSR and Adjacent Countries*. 4th edition. Israel Program for Scientific Translation, Jerusalem.  Wheeler, A. (1969). *Fishes of the British Isles and Northwest Europe.* Michigan State University Press, East Lansing, USA. |
| *Proterorhinus marmoratus* | Fecundity | Maitland, P.S. (2001). *Guide to Freshwater Fish of Britain and Europe.* Hamlyn, London, UK.  Miller, P.J. (2004). *The Freshwater Fishes of Europe - Volume 8/II: Gobiidae 2*. Aula, Wiebelsheim. |
|  | Egg Diameter | Miller, P.J. (2004). *The Freshwater Fishes of Europe - Volume 8/II: Gobiidae 2*. Aula, Wiebelsheim. |
| *Pylodictis olivaris* | Egg Diameter | Simon, T.P. & Wallus, R. (2004). *Reproductive Biology and Early Life History of Fishes in the Ohio River Drainage, Volume 3: Ictaluridae- Catfish and Madtoms.* CRC Press, New York, New York, USA. |
| *Scaphirhynchus platorhynchus* | Egg Diameter | Mitton, J.B. & Lewis Jr., W.M. (1989). Relationships between genetic variability and life-history features of bony fishes. *Evolution*, **43**, 1712-1723. |
|  |  |  |
